# Epitope‐based peptide vaccine design and target site depiction against Middle East Respiratory Syndrome Coronavirus: an immune-informatics study

**DOI:** 10.1186/s12967-019-2116-8

**Published:** 2019-11-08

**Authors:** Muhammad Tahir ul Qamar, Saman Saleem, Usman Ali Ashfaq, Amna Bari, Farooq Anwar, Safar Alqahtani

**Affiliations:** 1 College of Informatics, Huazhong Agricultural University, Wuhan, People’s Republic of China; 2 Department of Pharmaceutical Chemistry, College of Pharmacy, Prince Sattam bin Abdul Aziz University, Alkharj, Saudi Arabia; 3 Department of Bioinformatics and Biotechnology, Government College University Faisalabad, Faisalabad, Pakistan; 4 Department of Chemistry, University of Sargodha, Sargodha, Pakistan

**Keywords:** MERS-COV, Spike protein, T-and B-cell epitopes, Computational approaches, Vaccine design

## Abstract

**Background:**

Middle East Respiratory Syndrome Coronavirus (MERS-COV) is the main cause of lung and kidney infections in developing countries such as Saudi Arabia and South Korea. This infectious single-stranded, positive (+) sense RNA virus enters the host by binding to dipeptidyl-peptide receptors. Since no vaccine is yet available for MERS-COV, rapid case identification, isolation, and infection prevention strategies must be used to combat the spreading of MERS-COV infection. Additionally, there is a desperate need for vaccines and antiviral strategies.

**Methods:**

The present study used immuno-informatics and computational approaches to identify conserved B- and T cell epitopes for the MERS-COV spike (S) protein that may perform a significant role in eliciting the resistance response to MERS-COV infection.

**Results:**

Many conserved cytotoxic T-lymphocyte epitopes and discontinuous and linear B-cell epitopes were predicted for the MERS-COV S protein, and their antigenicity and interactions with the human leukocyte antigen (HLA) B7 allele were estimated. Among B-cell epitopes, QLQMGFGITVQYGT displayed the highest antigenicity-score, and was immensely immunogenic. Among T-cell epitopes, MHC class-I peptide YKLQPLTFL and MHC class-II peptide YCILEPRSG were identified as highly antigenic. Furthermore, docking analyses revealed that the predicted peptides engaged in strong bonding with the HLA-B7 allele.

**Conclusion:**

The present study identified several MERS-COV S protein epitopes that are conserved among various isolates from different countries. The putative antigenic epitopes may prove effective as novel vaccines for eradication and combating of MERS-COV infection.

## Background

Middle East Respiratory Syndrome-Coronavirus (MERS-COV), an extremely fatal respiratory infection was identified in 2012, when more than 90 cases were reported around the globe [1]. Since then, MERS-COV keeps on being a danger to worldwide human health and reported in 27 other countries including Jordan, Qatar, Germany, United Kingdom, Italy, Tunisia and France [2]. As of December-2018, total 2266 laboratory affirmed cases and 804 deaths with approximate 35.5% primitive–case casualty rate was accounted by world health organization (WHO). Solely Saudi Arabia were reported major figures of 1888 cases and 730 deaths [3].

The incubation period for MERS-COV is approximately 5 or 6 days and the fatality rate is ~ 30 to 40% [4]. Patients with severe acute respiratory illness caused by MERS-COV infection exhibit symptoms like coughing, fever, shortness of breath, diarrhoea, nausea/vomiting, highly lethal pneumonia, and kidney infection in most severe forms [5]. MERS-COV can create acute respiratory distress syndrome (ARDS) and have a higher chance of patient’s death from multi-organ failure, stubborn hypoxaemia and septic stun [6]. According to recent research, people with comorbidities including chronic lung disease, heart and kidney disease, cancer and diabetes are more likely to become infected with MERS, people with a weakened immunity system are also at higher danger of infection [3, 7]. Various mammalian and avian hosts can be infected with coronaviruses causing respiratory, enteric, hepatic or neurological diseases [8], and animals exposure with MERS-COV include camels, marmosets and macaques [9].

**Fig. 1:**
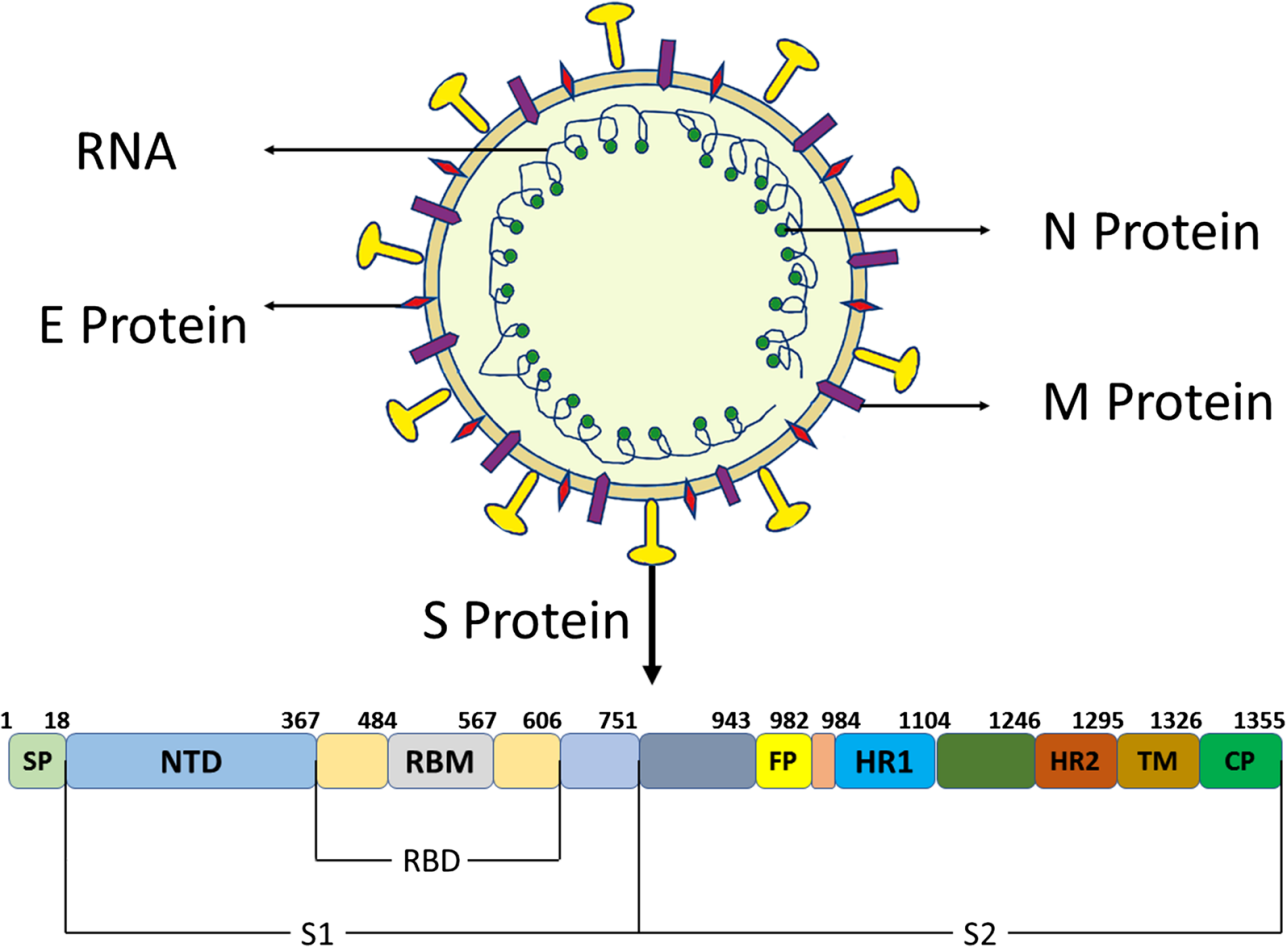
Diagram of MERS-COV genome encoding envelope spike (S) protein. S contains S1 and S2 subunits; *SP* signal peptide, *NTD* N terminal domain, *RBD* receptor-binding domain, *RBM* receptor-binding motif, *FP* fusion peptide, *HR1 and HR2* heptad repeat region 1 and 2; *TM* transmembrane and *CP* cytoplasmic tail regions

MERS-COV is caused by a novel single stranded, positive (+) sense RNA beta-coronavirus, which is a pathogen of zoonotic reservoir [9, 10]. The precise method of MERS-COV transmission is unrevealed, though dromedary camels [5, 9], and insectivorous bats are considered to be transmission hosts according to the current evidences [2, 3]. Among all RNA viruses, positive-sense single-stranded RNA coronaviruses possess the largest genome (28–32 kb) [9]. MERS-COV invades into host cells by attaching to specific di-peptidyl peptide receptors. On the host cell surface, the viral envelope-anchored spike (S) protein binds to its receptor leading to COV entrance into the host cells, and S protein’s protease cleavage is necessary for virus cell unification and the entrance of genomic RNA into the cytoplasm [3, 9]. The S protein (Fig. 1) includes the receptor binding S1-subunit and the membrane fusion S2-subunit. The host receptor specifically recognises by S1-subunit receptor-binding domain (RDB) and this RDB/receptor connection is the most crucial determinant of COV-host range. Virus infection raises the quantity of replication mistakes in the host genome [8]. Transcription and replication of viral RNA takes place on two fold layer vesicles and different membranous structures obtained from endoplasmic-reticulum [9, 11]. Sub-genomic negative (−) strand RNA intermediates are responsible for transcription of seven sub-genomic mRNA species [9, 11]. At the 5′ end of the genome, a common leader is encoded and attached to the 3′-terminus of sub-genomic RNA, and viral RNA is carried to the endoplasmic-reticulum Golgi -intermediate-compartment (ERGIC), which is the position of assembly and encapsulation of the N protein. Viral RNA is then transferred into vesicles-lined S, E and M proteins. Before secretion, vesicles are moved to the cell-surface. By infecting the same host cell, different COV particles can also recombine their genomes, resulting in rapid evolution. Thus, COVs can readily modify to various hosts, and frequently cross the species hindrances to expand host diversity [3, 9, 11].

At present, no specific therapeutic agent or vaccine is available on the market for the treatment of MERS infections [5]. Inhibition of MERS-COV by type-I interferons (IFNα and especially IFNβ) has been proposed based on experiments on cultured cells; lung injury can be reduced by a combination of ribavirin and IFNα2b, and within 8 h of virus immunization the lung titre is decreased in rhesus macaques [9, 12]. Developing an effective treatment for MERS is therefore a research priority. To this end, immuno-informatics can be applied for deep analysis of viral antigens, forecast of conformational (discontinuous) and linear epitopes, evaluation of immunogenicity, and virulence of pathogens. Furthermore, an immuno-informatics approach may save time and cost when designing novel vaccines against viruses, and the use of kits and related antibodies can be reduced [13, 14]. Therefore, using this approach, the main aim of the current study was to identify potential B- and T-cell epitope(s) based on envelope and nucleocapsid proteins that could be used to develop promising vaccines [15]. Extreme respiratory infection may also be recovered by T-cell and antibody reactions [12]. In addition, fast recognition and isolation, disease prevention, and control steps are crucial for preventing the MERS-COV transmission in households, communities, and healthcare offices [16, 17]. The main aim of the current study was to identify the potential B-cell and T-cell epitope(s) from the envelope S protein that could be used as promising vaccines agents against MERS-COV.

## Methods

### Data retrieval and structural analysis

Primary sequence of Saudi Arabia isolate MERS-COVS protein was retrieved from NCBI database using accession number ALW82742.1 [18]. Experimentally known 3D structure of MERS-COV S protein was retrieved by using PDB ID: 5X59 from Protein-Data-Bank [19]. Protein sequence was analysed for its chemicals and physical properties including GRAVY (Grand average of hydropathicity), half-life, molecular weight, stability index and amino acid atomic composition via an online tool Protparam [20]. Secondary structure of MERS-COV S protein was analysed through PSIPRED [21]. TMHMM an online tool (http://www.cbs.dtu.dk/services/TMHMM/), used to examine the transmembrane topology of S protein. Existence of disulphide-bonds were examined through an online tool DIANNA v1.1. It makes prediction based on trained neural system [22]. Antigenicity testing carried out through vaxijen v2.0 [23]. Allergenicity of query sequence was checked through AllerTOP v2.0 [24].

### B-cell epitope prediction

Freely online accessible servers IEDB (Immune-Epitope-Database And Analysis-Resource) [25] and BCPRED [26] were used to for B-cell epitopes forecast. Criteria was set to have 75% specificity and 14 residue lengthy epitopes were viewed as adequate to persuade defensive immune reaction. Only those epitopes were chosen that were visible on outer surface and other intracellular epitopes were eliminated. Vaxijen 2.0 server was utilized for antigenicity study of chosen epitopes [23]. Recognition of B-cell epitopes was depended on; antigenicity, accessibility of surface, flexibility, hydrophilicity and predictions of linear epitope [27]. Hydrophilicity, isolation of linear epitope, accessibility of surface and Flexibility analysis were performed through Bepipred linear epitope prediction and Parker hydrophilicity prediction algorithms, Kolaskar and Tongaonkar antigenicity scale, Emini surface accessibility prediction tool and Karplus and Schulz flexibility prediction tool [28]. Forecast of beta turns in polyprotein was done by utilizing Chou and Fasman beta-turn prediction algorithm [29]. As the discontinuous epitopes are increasingly explicit and have higher dominant attributes over linear epitopes [30, 31], so, the forecast of discontinuous epitopes have additionally been carried out via DiscoTope server [32]. Parameter was set at ≥ 0.5 which indicated 90% specificity and 23% sensitivity. This method relies on surface accessibility and amino acid statistics in a collected form dataset of discontinuous epitopes found out by X-ray crystallography of antigen/antibody protein buildings. At last, position of predicted epitopes clusters (positional affirmation) on 3D structure of S protein was observed via PepSurf [32]. Pymol was utilized to examine the positions of forecast epitopes on the 3D structure of MERS-COV S protein [33].

### T-cell epitope prediction

Cytotoxic T-lymphocyte (CTL) epitopes play a crucial role in vaccine designation. Most significant, it decreases the cost and time as compared with wet lab experiments [34]. By utilizing two distinctive online accessible tools Propred-1 [35] and Propred tool [36], CTL epitopes of target protein of MHC class-I and MHC class-II were predicted respectively. The outcomes of these tools are quite substantial because they utilize vast number of alleles of HLAs (human-leukocyte-antigens) during computation. The sequence was given in plain format and all alleles were chosen for prediction. For propred-1 proteasome and Immuno-proteasome filters with a threshold value of 5% were kept on.

### Eminent features profiling of selected T cells epitopes

After finalizing the epitopes of both MHC class-1 and MHC class-II alleles, their important features including digestion, mutation, toxicity, allergenicity, hydro and physiochemical were checked via vaxijen 2.0 [23], protein digest server (http://db.Systemsbiology.net:8080/proteomicsToolkit/proteinDigest.html), AllergenFP 1.0 [37] server, Aller Hunter server (https://omictools.com/allerhunter-tool) and ToxinPred server (http://crdd.osdd.net/raghava/toxinpred/). AllergenFP 1.0 is generally utilized for the prediction of allergenicity of epitopes for vaccine development [37]. Aller Hunter server compares peptide’s query sequences opposed to the database of previously reported allergens to give significant outcomes. An in silico method, ToxinPred is used to predict Non-Toxic/Toxic peptides. For further analysis, only NonToxic epitopes were chosen.

### Conservation analysis of selected epitopes

S protein sequences of 8 distinctive countries were taken from an open access Genbank database [38]. By utilizing CLC work bench, the multiple-sequence-alignment (MSA) was carried out to perceive the conservation of chosen epitopes [39]. The aligned files (.aln) were additionally utilized to make phylogenetic tree via MEGA7 software [40]. By analysing the multiple-sequence-alignment results and with IEDB conservation-analysis-tool, all the chosen epitopes were checked for their variability and conservation.

### Structural modelling and molecular docking

All the predicted peptides 3D structures were modelled via PEPFOLD server at RPBS MOBYL portal [41], from Protein databank (PDB ID: 3VCL) at a resolution of 1.7 Å, the 3D structure of human HLA-B7 allele crystallized was taken [42] and utilized for further molecular docking purpose. Through Molecular Operating Environment (MOE) tool, the peptide models (antigenic determinants) were docked against their respective HLA-B7 allele to analyse their inhibitory potential. Procedure for molecular docking using MOE has already been described in various studies [13, 43, 44]. Docking procedure utilized in those studies include protonation, expulsion of already bound peptide and energy reduction followed by expulsion of water particles. Triangular matcher algorithm was applied as default peptide placement methods dependent on the receptor shape which without energy optimization rapidly produces 1000 best poses of docked peptide [13]. By applying London-dG scoring function, the energy approximation of the imitated poses was rescored. For every peptide, top ten positioned poses of London-dG were additionally reduced by Force field refinement algorithm. Protein peptide connection were than examined via LigX tool of MOE. UCSF Chimera and Pymol tools were utilized to produce figures of docked complexes [33, 45].

## Results

### Structural analysis

The physiochemical properties of MERS-COV S protein computed via protparam demonstrates that it contained 1353 amino acids (aa) with molecular weight of 149,479.23 kDa, which reflects good antigenic nature. Theoretical isoelectric point (PI) of subject protein was 5.80 which indicate its negative in nature. An isoelectric point under 7 shows negatively charged protein. Briefly, out of 1353 residue, 112 aa were found as negatively charged whereas others found as positively charged. Protparam computed instability-index (II) 36.81, this categories protein as stable. Aliphatic-index 82.79, which devotes a thought of proportional volume hold by aliphatic side chain and GRAVY value for protein sequence is 0.078. Half-life of protein depicted as the total time taken for its vanishing after it has been synthesized in cell, which was computed as 30 h for mammalian-reticulocytes, > 20 h for yeast, > 10 h for *Escherichia coli*. Total number of Carbon (C), Oxygen (O), Nitrogen (N), Hydrogen (H) and Sulfur (S) were entitled by formulaC_6687_H_10258_N_1740_O_2027_S_63_. Protparam computed details of physiochemical properties enlisted in Additional file 2: Table S1.

Secondary and 3-D structure examination of S protein via PSIPRED [21], UCSF Chimera [45] and Pymol [33] respectively showed that (50%) Beta sheets, (10%) Helixes and (40%) Loops are present in structure as shown in Additional file 1: Figure S1. Two different conformations of structure of MERS-COVS protein shown in Additional file 1: Figure S2.

Furthermore in target protein, DiANNA1.1 tool [22] calculated 21 disulphides bond (S–S) positions and assign them a score given in Additional file 2: Table S2. Antigenicity of protein was evaluated via Vaxijen 2.0 [23] by setting the threshold at ≥ 0.5, for higher specificity. Antigenicity analysis of full-length protein showed antigenicity 0.4808 for S protein showing it as an expected antigen. An online tool TMHMM used to checked the transmembrane protein topology (http://www.cbs.dtu.dk/services/TMHMM/) and it was found that residue from 1 to 1295 were exposed on the surface, while residue from 1296 to 1318 were inside transmembrane-region and residues from 1319 to 1353 were buried within the core-region of the S protein.

### Recognition of B-cell epitopes

B-cell epitopes are significant for defence against viral disease. Potential B-cell epitopes have different features that direct B-cell to recognize and activate the rich defence responses against distinct viral infection. Primary sequence of S protein was scanned via IEDB server [25] and BCPRED [26] to predict B-cell epitopes. Total 59 B-cell epitopes were predicted. From all predicted epitopes, just6 epitopes (Table 1) were selected which were exposed on the surface of S protein and have high antigenicity score. Vaxijen 2.0 was used to compute antigenicity score and TMHMM server was utilized to check the surface availability. Among these selected epitopes, ‘QLQMGFGITVQYGT’ predicted at position 566 showed highest antigenicity and predicted scores.

**Table 1:** B-cell epitopes present on surface predicted via IEDB analysis resource and BCPRED are shown along with their starting positions and antigenicity scores

Sr#	Position	Epitopes sequences	Score	Antigenicity
1	13	TPTESYVDVGPDSV	0.6484	0.938
2	209	TPATDCSDGNYNRN	0.6048	0.982
3	251	LEWFGITQTAQGVH	1.1063	0.845
4	566	QLQMGFGITVQYGT	1.5236	0.995
5	1287	GNYTYYNKWPWYIW	0.7180	0.884
6	1339	RYEEYDLEPHKVHV	1.3061	0.890

**Fig. 2:**
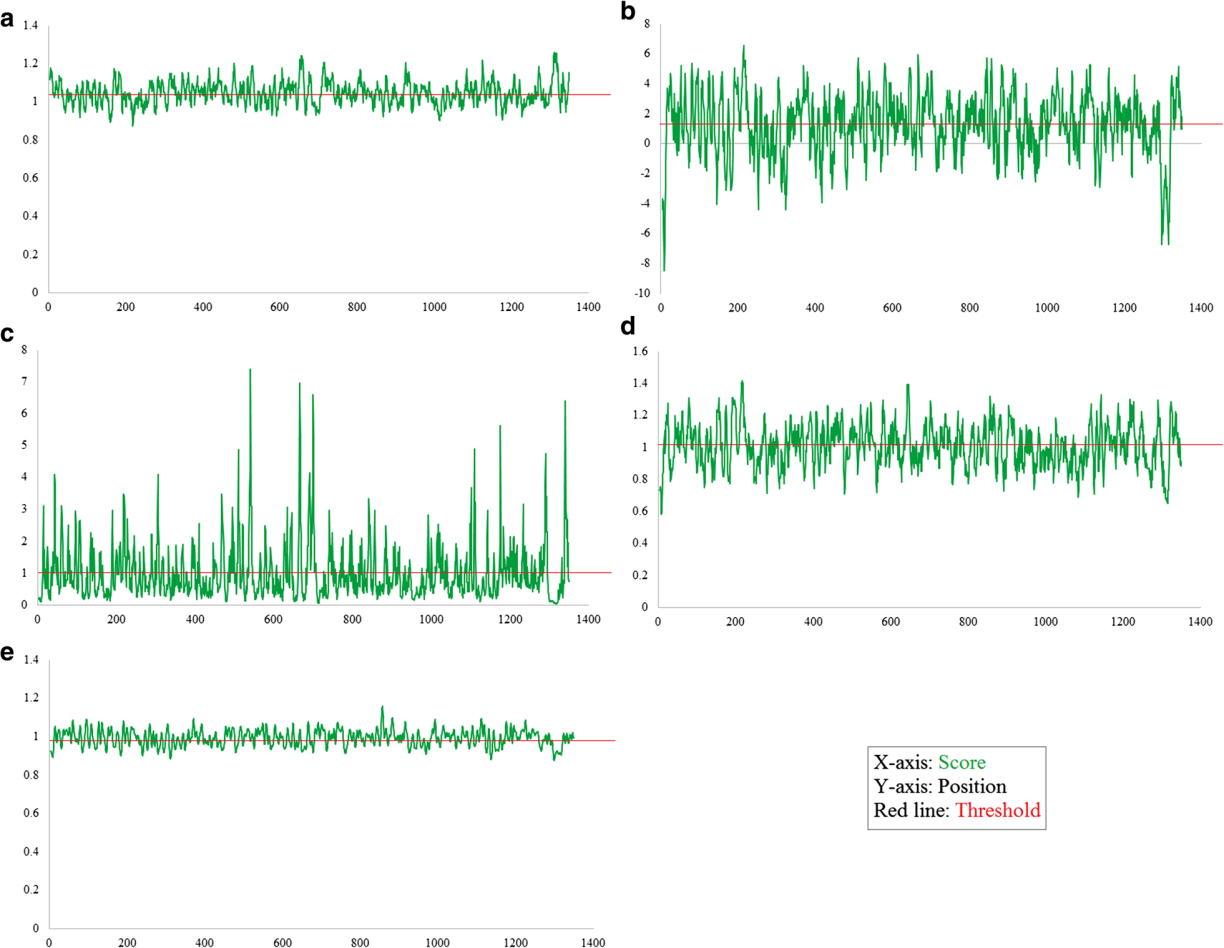
**a** Prediction of antigenic determinants using Kolaskar and Tongaonkar antigenicity scale, **b** hydrophilicity prediction using Parker hydrophilicity, **c** surface accessibility analyses using Emini surface accessibility scale, **d** beta turns analyses in structural polyprotein using Chou and Fasman beta turn prediction, **e** flexibility analyses using Karplus and Schulz flexibility scale

**Fig. 3:**
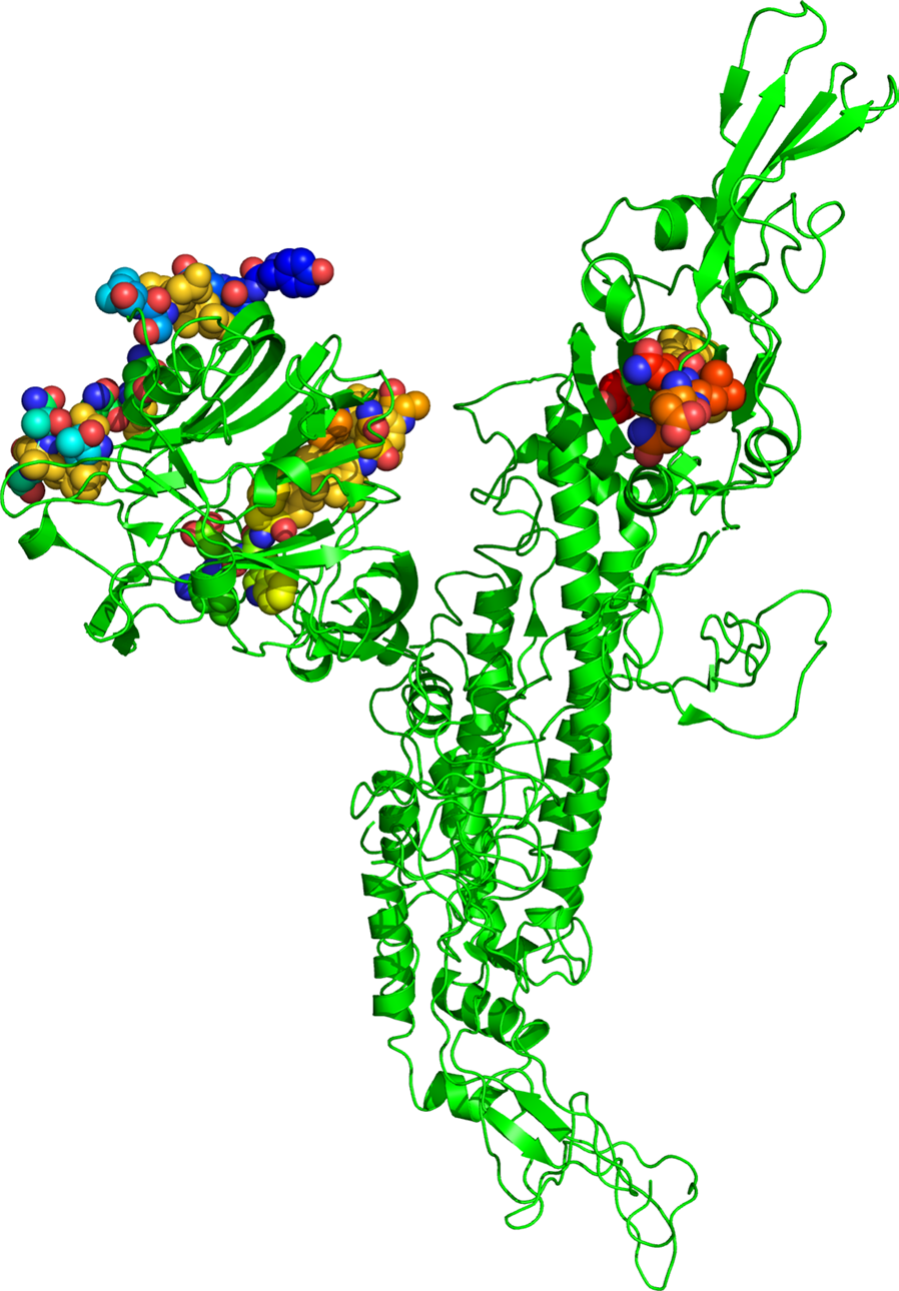
Site of B cells predicted linear epitopes on the crystal structure of MERS-COV envelope S protein highlighted through cartoon representation

Moreover, it is essential to check out the surface availability of possible B-cell epitopes. Kolaskar and Tongaonkar antigenicity measurement tools analysed the S protein for prediction of B-cell epitopes by assessing the physiochemical properties of the amino acid and their abundance in already known B-cell epitopes. Higher antigenicity score has proposed that it can play a vital role in starting of immune response. The threshold value of tool was adjusted at 1.045 and window size was kept 7. It estimated the antigenic tendency value of protein 1.045 (average), 0.872 (minimum) and 1.258 (maximum). Result of kolaskar and Tongaonkar analysis are shown in Fig. 2a. Hydrophilic region of protein is generally uncovered on the surface and play a significant part in eliciting the immune response. BCPRED-score and calculated antigenicity outcomes of vaxijen surely manifest that all predicted peptides are part of extracellular area of transmembrane-protein and capable to maximize a defence response inside the host during MERS-COV infection. Therefore, to find the surface availability of possible B-cell epitopes and hydrophilicity, parker-hydrophilicity with threshold value 1.279 and Emini surface accessibility prediction tools with threshold value 1.000 were utilized. The visual representation of outcome of both tools is shown in Fig. 2b, c respectively. Values calculated by both these tools were 1.279 (average), − 8.486 (minimum), 6.543 (maximum); and 1.000 (average), 0.033 (minimum), 7.392 (maximum), respectively. Emini surface accessibility analysing tool’s outcomes are given in additional file 2: table S3. Chou and Fasman beta turn analysing algorithm was utilized to predict beta-turn in S protein because beta turn is exposed on the surface and hydrophilic in nature and play a vital role in starting the defence response. Tool’s threshold was adjusted at 1.009, it computed the values which are 1.009 (average), 0.581 (minimum), and 1.414 (maximum). Chou and fasman’s result’s graphical representation is shown in Fig. 2d. The result indicates that region from 213 to 220 amino acid and from 641 to 650 are more disposed to persuade Bturns in peptide structure. It is described by an experimental information that the parts of epitope which connect with antibodies or alleles are mainly elastic in nature. Karplus and schulz flexibility analysing tool represented that the area from amino acid from 854 to 860 sequence positions are highly versatile as shown in Fig. 2e. Position of every predicted epitope on surface of 3-D structure of S protein was confirmed by Pepsurf [32] and shown in Fig. 3 using Pymol [33].

**Fig. 4:**
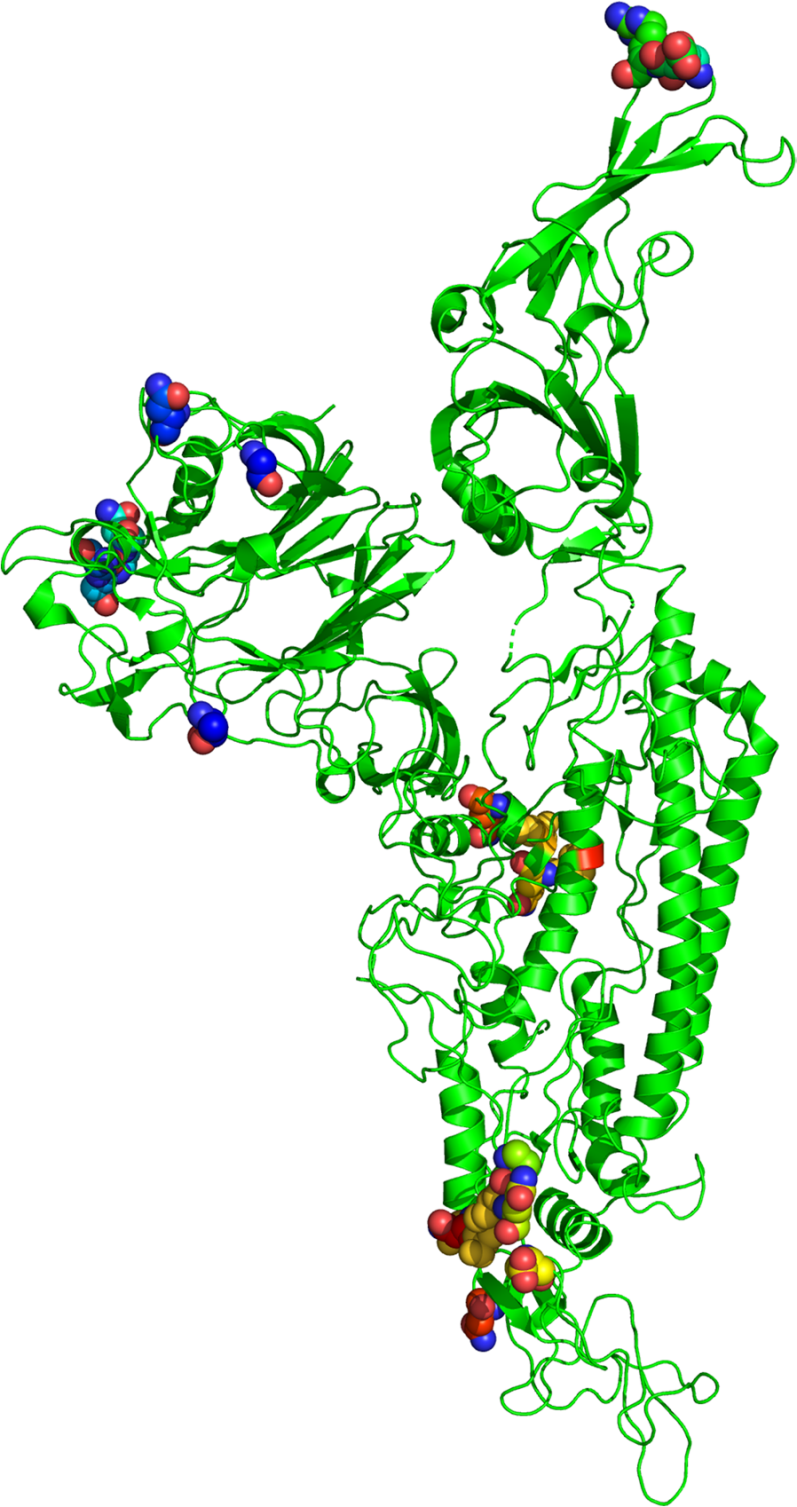
Site of B cells discontinues epitopes predicted through DISCOTOPE 2.0 Server on the crystal structure of MERS-COV envelope S protein highlighted with cartoon representation

To further increase the specificity and range of B-cell epitopes, Discotope 2.0 server was used which calculate surface availability in term of residue contact number and novel tendency amino acid score was utilized to predict the discontinuous epitopes. 3D structure of S protein (PDB ID: 5X59) [19] was used for discontinuous epitopes prediction, 90% specificity, − 3.700 threshold and 22.000 Angstroms propensity score radius. Total 22 discontinuous epitopes were calculated at different exposed surface areas (Table 2). Position of each predicted epitope on surface of 3D structure of S protein shown in Fig. 4 using Pymol [33].

**Table 2:** Discontinuous epitopes predicted through DISCOTOPE 2.0 Server

Sr#	Residues position	Residues names	Number of contacts	Propensity score	DiscoTope score
1	43	THR	0	− 4.114	− 3.641
2	159	GLY	2	− 2.029	− 2.026
3	199	ASN	1	− 2.667	− 2.591
4	215	SER	4	− 2.632	− 2.790
5	216	ASP	0	− 1.426	− 1.262
6	217	GLY	0	− 0.316	− 0.279
7	218	ASN	5	− 0.626	− 1.129
8	509	ASP	4	− 3.406	− 3.474
9	510	ASP	0	− 3.963	− 3.508
10	511	ARG	1	− 3.183	− 2.932
11	767	PRO	4	− 2.719	− 2.866
12	768	ILE	11	− 2.612	− 3.577
13	769	GLN	6	− 1.481	− 2.000
14	771	ASP	3	− 1.702	− 1.851
15	785	ASN	11	− 2.269	− 3.237
16	906	MET	1	− 2.453	− 2.286
17	907	GLN	1	− 3.101	− 2.859
18	910	ASP	3	− 3.381	− 3.337
19	914	GLN	7	− 2.405	− 2.933
20	993	ASN	6	− 1.388	− 1.918
21	1145	ASN	7	− 2.245	− 2.800
22	1146	HIS	13	− 2.206	− 3.288

Residues are shown in three-letter code, and number of contacts shows the connection of amino acid with others

### Recognition of T-cell epitopes

Propred-I (47 MHC class-I alleles) [35] and Propred (51 MHC class-II alleles) [36] were utilized for prediction of T-cell epitopes for the S protein. Propred-I utilizes a matrix base approach to scan and predict the peptides against library of 47 MHC class-1 alleles. The S protein sequence in FASTA format was transferred to the propred-I server, whereas choosing all the alleles with higher scoring peptide with 4% threshold and keeping the proteasome filter and immune proteasome filter at on mode. Additionally, antigenicity testing and screening of peptides were finished with assistance of vaxijen 2.0 [23]. Just 6 potential peptides were chosen for next processing on the basis of their antigenicity-score (Table 3). A peptide which has capacity to attach with larger number of alleles is observed as most important peptide due to its potential to bring a powerful defense response. Between MHC class-I predicted epitopes, the peptide ‘YKLQPLTFL’ indicated higher antigenicity score 1.5335 attaching with number of alleles including MHC-Db, HLA-Cw*0301, HLA-B*51, HLA-B*5401, HLA-B*5301, HLA-B*3902, HLA-B*3901, HLA-B*3701, HLA-B7, HLA-B14, HLA-A2.1, HLA-A20 Cattle, HLA-A2 and HLA-A*0201.

**Table 3:** MHC class-I allele binding peptides predicted via Propred-I with their antigenicity scores

Sr#	Peptides	MHC class-I alleles	Vaxijen score
1	YKLQPLTFL	MHC-Db, HLA-Cw*0301, HLA-B*51, HLA-B*5401, HLA-B*5301, HLA-B*3902, HLA-B*3901, HLA-B*3701, HLA-B14, HLA-A2.1, HLA-A20 Cattle, HLA-A2, HLA-A*0201, HLA-B7	1.3798
2	LTLLEPVSI	MHC-Kk, MHC-Kd, HLA-B*5801, HLA-B*51, HLA-B*5103, HLA-B*5301, HLA-B7	0.9748
3	ESAALSAQL	MHC-Ld, HLA-Cw*0602, HLA-B60, HLA-B40, HLA-B*3902, HLA-A*3302, HLA-B7	0.7965
4	IAGLVALAL	MHC-Db revised, HLA-B8, HLA-B7, HLA-B60, HLA-B*5801, HLA-B*5103, HLA-B*5102, HLA-B*5101, HLA-B*3501	0.7799
5	AGYKVLPPL	MHC-Dd, HLA-B7, HLA-B60, HLA-B*5401, HLA-B*5201, HLA-B*5103, HLA-B*5102, HLA-B*5101, HLA-B40, HLA-B*3901, HLA-B*3701, HLA-B*2705, HLA-A*0205, HLA-B14	0.6416
6	WPRPIDVSK	HLA-A*1101, HLA-A3, HLA-A68.1, HLA-B*5301, HLA-B*5401, HLA-B*51, HLA-B*0702, HLA-B7	0.6160

Propred, a quantitative matrix base method was used for prediction of peptides, which can interact with MHC class-II alleles. Sequence was given in FASTA format to Propred. Screening was done with the help of vaxijen 2.0 and just 6 high scoring epitopes were chosen (Table 4). The peptide ‘YCILEPRSG’ was considered more antigenic for its higher antigenicity score 1.7889 and it demonstrated virtual attachment with larger number of alleles (almost 15) including, DRB5_0105, DRB5_0101, DRB1_1328, DRB1_1327, DRB1_1323, DRB1_1307, DRB1_1305, DRB1_1302, DRB1_1301, DRB1_1128, DRB1_1120, DRB1_1114, DRB1_1101, DRB1_0802 and DRB1_0101.

**Table 4:** MHC class-II allele binding epitopes predicted using Propred with their antigenicity scores

Sr#	Peptides	MHC class-II alleles	Vaxijen score
1	YCILEPRSG	DRB5_0105, DRB5_0101, DRB1_1328, DRB1_1327, DRB1_1323, DRB1_1307, DRB1_1305, DRB1_1302, DRB1_1301, DRB1_1128, DRB1_1120, DRB1_1114, DRB1_1101, DRB1_0802, DRB1_0101	1.5044
2	LYFMHVGYY	DRB1_0301, DRB1_0802, DRB1_0806, DRB1_0817, DRB1_1104, DRB1_1106, DRB1_1128, DRB1_1305, DRB1_1311, DRB1_1321	1.4863
3	MRLASIAFN	DRB1_0301, DRB1_0306, DRB1_0307, DRB1_0308 DRB1_0311, DRB1_0405, DRB1_0410, DRB1_0423, DRB1_0801, DRB1_0802, DRB1_0804, DRB1_0806, DRB1_0817, DRB1_1101, DRB1_1102, DRB1_1104, DRB1_1106, DRB1_1107, DRB1_1114, DRB1_1120, DRB1_1121, DRB1_1128, DRB1_1301, DRB1_1302, DRB1_1304, DRB1_1305, DRB1_1307, DRB1_1311, DRB1_1321, DRB1_1322, DRB1_1323, DRB1_1327, DRB1_1328	1.4844
4	FGITQTAQG	DRB1_1321, DRB1_1307, DRB1_1305, DRB1_1128 DRB1_1101, DRB1_0801, DRB1_0426, DRB1_0421 DRB1_0401	1.3764
5	VRIGAAANS	DRB1_1328, DRB1_1327, DRB1_1322, DRB1_1301 DRB1_1121, DRB1_1107, DRB1_1102, DRB1_0426 DRB1_0402, DRB1_0401, DRB1_0311, DRB1_0308 DRB1_0307, DRB1_0306, DRB1_0301	1.1906
6	VYKLQPLTF	DRB5_0101, DRB5_0105, DRB1_1327, DRB1_1328 DRB1_1128, DRB1_1301, DRB1_0102, DRB1_1101, DRB1_1104, DRB1_1106, DRB1_1305, DRB1_1311	1.1127

### Eminent features profiling of selected T cells epitopes

Some important features of selected epitopes were analysed to support our findings. The peptides that can be digested by several enzymes are usually non-stable. On the other hand, peptides digested by fewer enzymes are highly stable and more favourable vaccine candidates. Peptides digesting enzymes were predicted through Protein digest server. Allergen FP 1.0 was used for allergenicity prediction of epitopes. ToxinPred was utilized for toxicity prediction of chosen epitopes. Toxinpred is based on support vector machine (SVM) used to predict toxicity along with mutations, hydropathicity, hydrophilicity, hydrophobicity, and charge. All T-cell epitopes along with their digestion, mutation, toxicity, allergenicity, hydro and physiochemical results are given in Table 5.

**Table 5:** Digestion, Mutation, toxicity, allergenicity, hydro and physiochemical profiling of selected peptides

Sr#	Peptides	Non-digesting enzymes	Mutation position	Toxicity	Allergenicity	Hydrophobicity	Hydrophilicity	Charge	PI	Mol. weight
MHC class-I binding peptides										
1	YKLQPLTFL	Trypsin R, Clostripain, IodosoBenzoate, AspN, Cyanogen Bromide, Staph Protease	NM	NT	NA	0.07	− 0.92	1.00	8.59	1122.37
2	LTLLEPVSI	Trypsin, Clostripain, AspN, Chymotrypsin, Cyanogen Bromide, IodosoBenzoate, Trypsin R, Trypsin K	NM	NT	NA	0.25	1.80	− 1.00	4.00	984.20
3	ESAALSAQL	Clostripain, Chymotrypsin, Cyanogen Bromide, AspN Trypsin K, IodosoBenzoate, Proline Endopept, Trypsin R	NM	NT	NA	0.05	− 0.31	− 1.00	4.00	888.97
4	IAGLVALAL	Trypsin, Staph Protease, AspN, Chymotrypsin, Trypsin R, Clostripain, Trypsin K, CyanogenBromide, IodosoBenzoate, Proline Endopept	NM	NT	NA	0.43	− 1.20	0.00	5.52	840.72
5	AGYKVLPPL	Clostripain, IodosoBenzoate, Staph Protease, AspN, Trypsin R, Cyanogen Bromide	NM	NT	NA	0.13	− 0.67	1.00	8.63	957.18
6	WPRPIDVSK	Trypsin, Chymotrypsin (modified)Chymotrypsin, Cyanogen Bromide, Trypsin K, Trypsin R, Staph Protease	NM	NT	NA	− 0.26	0.29	1.00	8.75	1097.28
MHC class-II binding peptides										
1	YCILEPRSG	Cyanogen Bromide, Trypsin K, AspN, IodosoBenzoate	NM	NT	A	− 0.11	− 0.06	0.00	5.99	1037.20
2	LYFMHVGYY	Trypsin, AspN, Clostripain, IodosoBenzoate, Staph Protease, Trypsin K, Trypsin R, Proline Endopept	NM	NT	NA	0.20	− 1.61	0.50	6.74	1192.40
3	MRLASIAFN	IodosoBenzoate, Trypsin K, AspN, Staph Protease, Proline Endopept	NM	NT	A	− 0.07	− 0.47	1.00	9.50	1022.23
4	FGITQTAQG	Trypsin, AspN, Clostripain, Cyanogen Bromide, IodosoBenzoate, Trypsin R, Proline Endopept, Staph Protease, Trypsin K	NM	NT	NA	0.02	− 0.58	0.00	5.52	922.01
5	VRIGAAANS	Chymotrypsin, Trypsin K, IodosoBenzoate, Proline Endopept, Staph Protease, Cyanogen Bromide, AspN	M	NT	A	− 0.07	− 0.10	1.00	9.72	857.97
6	VYKLQPLTF	AspN, Trypsin R, Clostripain, Staph Protease, Cyanogen Bromide, IodosoBenzoate	NM	NT	NA	0.02	− 0.79	1.00	8.56	1108.35

*NM* no mutation, *NT* non-toxic, *T* toxic, *NA* non-allergic, *A* allergic and the non-digesting enzymes showing those enzymes which do not digest peptides into fragments

### Conservation analyses of selected epitopes

**Fig. 5:**
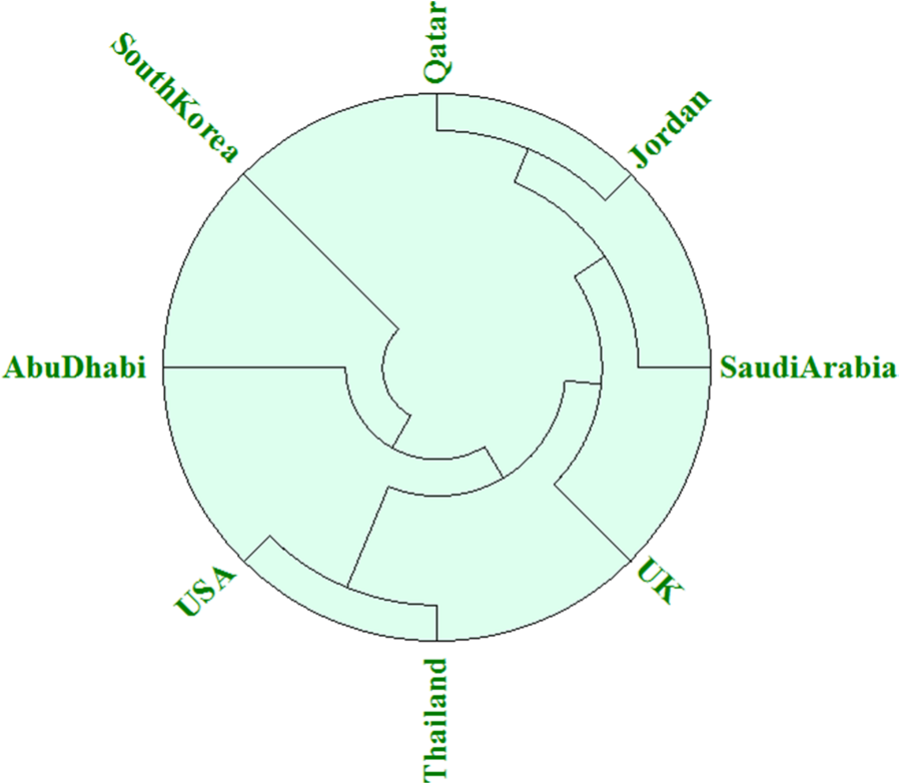
Phylogenetic tree illustrating evolutionary relationships among MERS-COV isolates of 8 different countries

Sequence of MERS-COV S protein from 8 different countries isolates including Saudi Arabia (ALW82742.1), Abu Dhabi (ASU90340.1), Jordan (ASY99842.1), Qatar (AHX71946.1), South Korea (AKL59401.1), Thailand (ALD51904.1), United Kingdom (AJD81440.1) and United State (AHZ58501.1) were subjected to multiple-sequence-alignment through CLC workbench to analyse the conservation of chosen epitopes. It was noticed that all the chosen epitopes are mostly conserved in all sequences utilized for analysis as shown in Additional file 1: Figure S3. A phylogenetic tree was created to indicate the evolutionary relationship of MERS-COV of 8 distinct countries as shown in Fig. 5.

The epitope-conservancy study through IEDB epitope conservancy analysis tool shows that all of selected B-cell and T-cell (MHC class-I and II) epitopes have 100% identity and conserved in all isolates of distinct countries (Additional file 2: Table S4).

### Interaction study of predicted peptides with HLA alleles

3D structures of all 6 MHC class-I attaching peptides were predicted via PEPFOLD [41]. It created 5 models of every peptide; one best model was chosen for every peptide (Additional file 1: Figure S4). At first models were refined via energy minimization in MOE and peptide library involved of 6 peptides was made to dock with explained structure of HLA-B7 allele.

**Fig. 6:**
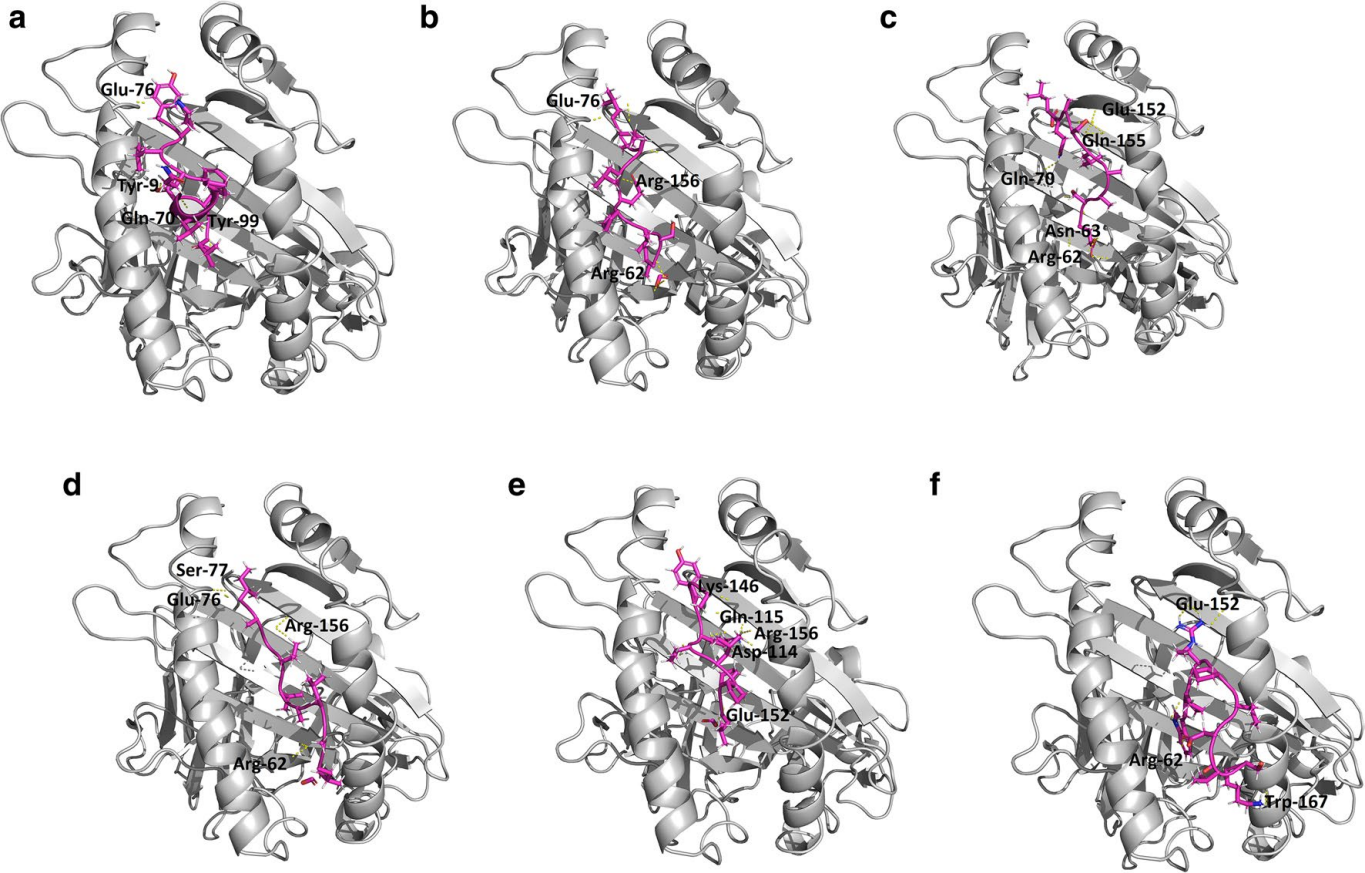
Graphical representation of interaction analyses between human HLA-B7 protein (shown in grey) and MHC class-I alleles binding peptides (shown in purple). The figure is in symmetry with the information provided in Table 6 and showing the interacting residues

Crystal structure of human HLA-B7 (PDB ID: 3VCL) protein was previously accessible with co-crystallized peptide in PDB [42]. So, rigid/focused docking was performed by utilizing same active pocket to dock our peptide library. 10 confirmations for every epitope were produced and top positioned conformations dependent on their dock scores and interactions with HLA-B7 residues were chosen (Table 6). Afterward, interaction examination by ligX tool of MOE was done (additional file 1: figure S5) which displayed that the peptide ‘AGYKVLPPL’ with highest dock score (-20.9793 kcal/mol) is connecting with key catalytic residues. Human HLA-B7 is a hetero-dimer structure, from the interaction analysis it was showed that Asp-114, Gln-115, Lys-146, Glu-152 and Arg-156 from A chains were making stable hydrogen bonds with the previously mentioned peptide (Fig. 6a). Peptide ‘WPRPIDVSK’ was docked (dock score -20.4007 kcal/mol) inside the catalytic pocket of receptor protein through 4 hydrogen bonds with Arg-62, Glu-152, Glu-163 and Trp-167 (Fig. 6b). Peptide ‘ESAALSAQL’ has -19.9914 kcal/mol of dock score with 5 stable hydrogen bonds between peptide and Arg-62, Asn-63, Gln-70, Glu-152 and Gln-155 (Fig. 6c). Similarly, other peptides also show strong and stable bonding with human HLA-B7 residues and shown in Table 6 and Fig. 6d–f.

**Table 6:** Molecular docking results of HLA-B7 with MHC class-I binding peptides have been given

Sr.	MHC class-I binding peptides	Docking score	Interacting residues
a	YKLQPLTFL	− 19.1695	Tyr-9, Gln-70, Glu-76, Tyr-99
b	LTLLEPVSI	− 19.3901	Arg-62, Glu-76, Arg-156
c	ESAALSAQL	− 19.9914	Arg-62, Asn-63, Gln-70, Glu-152, Gln-155
d	IAGLVALAL	− 19.0437	Arg-62, Glu-76, Ser-77, Arg-156
e	AGYKVLPPL	− 20.9793	Asp-114, Gln-115, Lys-146, Glu-152, Arg-156
f	WPRPIDVSK	− 20.4007	Arg-62, Glu-152, Glu-163, Trp-167

MOE was used for these analyses

## Discussion

Emergence of new viral diseases in resource poor countries in Asia represent a huge global disease burden. The population of developing countries such as Saudi Arabia is facing a serious health threat from MERS-COV virus, and there is an urgent need for corresponding therapies and preventative measures. MERS syndrome is characterised by lung and kidney infections [46]. This virus undergoes rapid evolution due to recombination between genomes of different viral particles after infecting host cells. At present, there are no reliable, specific drugs against MERS-COV infection available on the market [47].

Medical biotechnology is playing a significant role in the development of vaccines against these and similar viruses, but computer-based immune-informatics can be used for analysis of immunogenic data and vaccine development, and this approach can decrease time and cost. The specificity of epitope-based vaccines can be enhanced by only selecting the antigenic parts of proteins exposed on the surface, since these elicit strong immune responses [48, 49]. The viral S protein is considered a primary target for neutralising antibodies, and the S1 subunit of the S protein has been the focus of immunisation strategies to overcome MERS-COV disease [50]. The MERS-COV S protein is an immunogenic protein that plays an important role in the attachment and entry of viral particles in host cells, characterised by high antigenicity and surface exposure [51].

Herein, we explored epitope-based vaccine development targeting S protein potential B- and T-cell S protein epitopes that may promote an immune response in the host were identified, analyses were performed at protein primary, secondary and tertiary structural levels. B-cell conserved epitopes (≥ 14 residues long) were predicted by IEDB analysis-resource and BCPRED. Other tools in IEDB were utilised to analyse antigenicity, flexibility, solvent accessibility and disulphide bonds. The ‘QLQMGFGITVQYGT’ yielded a higher immunogenicity score (1.5236) and may represent a potential B-cell epitope and vaccine candidate. In addition, several T-cell antigenic determinants possessing the ability to bind MHCI and/or MHCII were predicted using Propred-I and Propred, respectively. MHC-I (YKLQPLTFL) and MHC-II (YCILEPRSG) epitopes interact with numerous HLA alleles and are highly antigenic in nature [52]. In addition, the positions of all predicted epitopes on the 3D structure were confirmed using Pepsurf. Discotope servers were used to predict discontinuous epitopes. Among MERS-COV strains, conservation of predicted epitopes from different countries was analysed to select epitopes common to all. The immune-informatics approach can identify highly conserved epitopes that may deliver wide protection against different strains. Conservation assessment revealed that all predicted epitopes were conserved between MERS-COV gene sequences reported from eight countries. Furthermore, allergenicity, toxicity, mutation and physiochemical properties of predicted antigen determinants were analysed to further increase specificity and selectivity. Digestion analysis confirmed that peptides identified in this study were stable and safe to use. On the basis of immunogenicity score and sequence conservation, it is clear that the conserved peptides are likely to be immunogenic. In addition, 3D structures of all six MHC class I binding peptides were predicted via PEPFOLD and docked with the human HLA-B7 allele by MOE to analyse binding specificity and defence response. Based on docking score, binding potential to HLA-B7, and immunogenicity score, peptides identified in the current study may prove highly immunogenic compared with previously reported peptides [51, 53, 54]. The predicted epitopes should be tested for therapeutic potency in future studies. We predict that the putative epitopes may have therapeutic potential with excellent scope. Our immune-informatics analysis identified potential strong T- and B-cell epitopes that may assist the development of potent peptide-based vaccines to address the imminent MERS-COV challenge.

## Conclusions

In the present study, a reverse vaccinology approach was adopted to identify surface-exposed peptides, rather than focus on the whole pathogen, which is a less efficient and effective process. This approach can reduce time and cost, and increase specificity. Only immunogenic regions of antigenic epitopes of the S protein of MERS-COV were screened to identify potential vaccine candidates. Sequence, structure, conservation and interaction analyses were conducted to discover epitopes of B- and T-cells that are antigenic and conserved among MERS-COV isolates from eight different countries, that may serve as vaccine candidates. The small number of antigenic epitopes identified in this study might deliver a preliminary set of epitopes for future vaccines against MERS-COV, which may help to control this growing health threat.

## Supplementary information

**Additional file 1: Figure S1**. PSIPRED analysis of the MERS-COV S protein. Helixes are cylindrical and coloured pink, beta-strands are shown as arrows and coloured yellow, and random coil regions are black. **Figure S2**. The 3D structure of the MERS-COV S protein (front and back conformations). **Figure S3**. Multiple sequence alignment showing conservation of the S protein of MERS-COV isolated from eight distinct countries. **Figure S4**. 3D (A1–6) and stick structures (B1–6) representation of selected MHC class-I alleles binding peptides. The figure is in symmetry with the information provided in Table 3. **Figure S5**. 2D graphical representation of interaction analyses between human HLA-B7 protein and MHC class-I alleles binding peptides. The figure is in symmetry with the information provided in Table 6 and Fig. 6 and showing the residues interacting with strong hydrogen bonding.

**Additional file 2: Table S1**. Physico-Chemical parameters of spike (S) protein computed through ExPASy ProtParam server**. Table S2**. Predicted disulphide bonds within residues of S protein via DiANNA 1.1 web Server. The bonds with lowest Score indicated as red colours are weak bonds. **Table S3**. Emini surface accessibility prediction results computed through IEDB Analysis Resource**. Table S4**. Conservancy results of B-cells and T-cells (MHC Class-I and II) epitopes among all 8 MERS-CoV isolates of distinct countries (Saudi Arabia, Abu Dhabi, Jordan, South Korea, Qatar, Thailand, USA and UK) have been shown. The analyses were done utilizing the IEDB Analysis Resource.

## Data Availability

Not applicable.

## Abbreviations

MERS-COV: Middle East Respiratory Syndrome Coronavirus
S: spike
RDB: receptor-binding domain
NCBI: National Centre for Biotechnology Information
PDB: Protein Data Bank
PI: isoelectric point
MHC: major histocompatibility complex
