# Supplementary material for: Epitope‐based peptide vaccine design and target site depiction against Middle East Respiratory Syndrome Coronavirus: an immune-informatics study
Source: J Transl Med. 2019 Nov 8;17:362. doi: 10.1186/s12967-019-2116-8 (PMC6839065; doi:10.1186/s12967-019-2116-8)
Supplement: Supplementary file 1 — Additional file 1: Figure S1. PSIPRED analysis of the MERS-COV S protein. Helixes are cylindrical and coloured pink, beta-strands are shown as arrows and coloured yellow, and random coil regions are black. Figure S2. The 3D structure of the MERS-COV S protein (front and back conformations). Figure S3. Multiple sequence alignment showing conservation of the S protein of MERS-COV isolated from eight distinct countries. Figure S4. 3D (A1–6) and stick structures (B1–6) representation of selected MHC class-I alleles binding peptides. The figure is in symmetry with the information provided in Table 3. Figure S5. 2D graphical representation of interaction analyses between human HLA-B7 protein and MHC class-I alleles binding peptides. The figure is in symmetry with the information provided in Table 6 and Fig. 6 and showing the residues interacting with strong hydrogen bonding. [file 12967_2019_2116_MOESM1_ESM.docx]

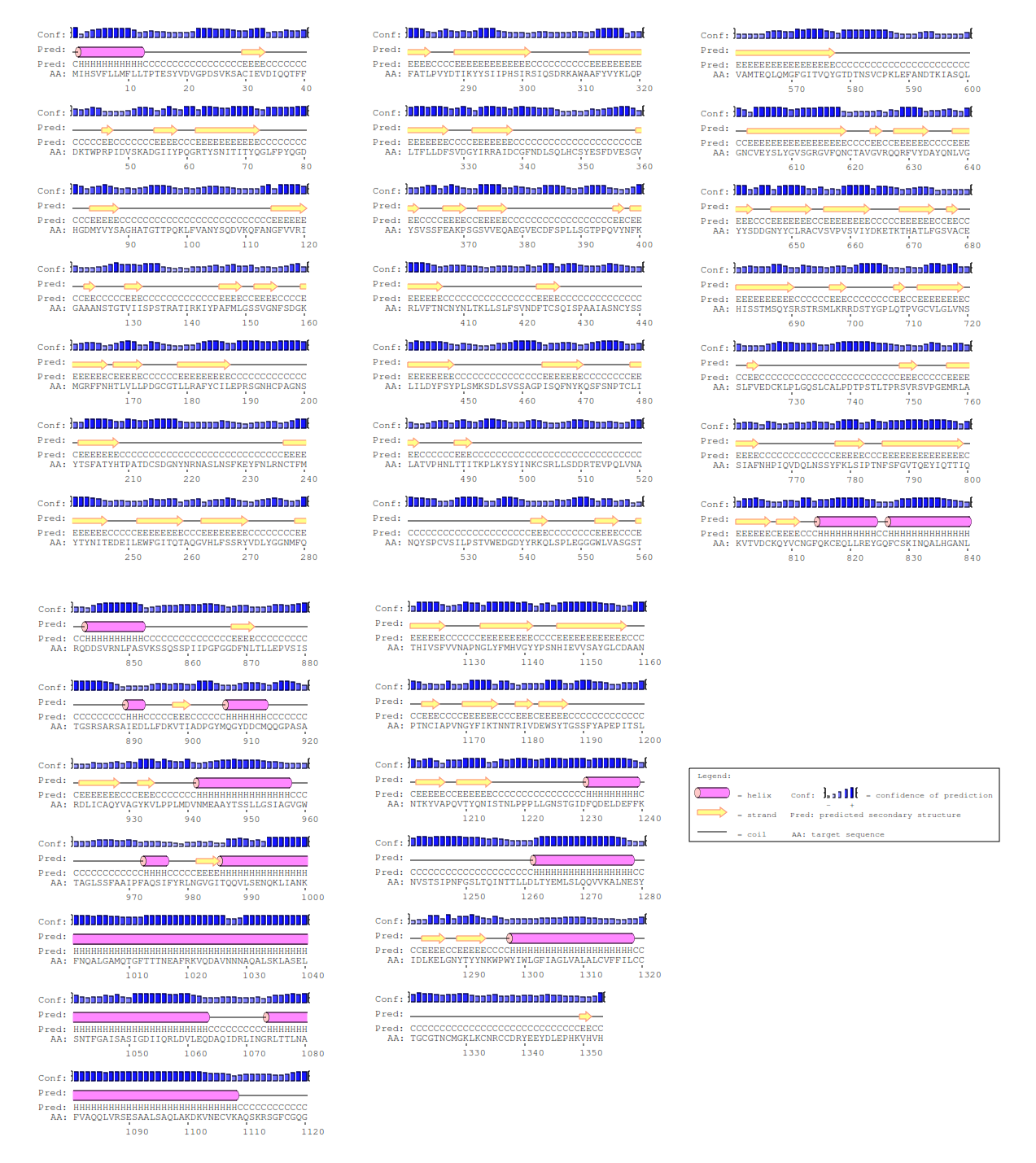


**Figure S1**. PSIPRED analysis of the MERS-COV S protein. Helixes are cylindrical and coloured pink, beta-strands are shown as arrows and coloured yellow, and random coil regions are black.


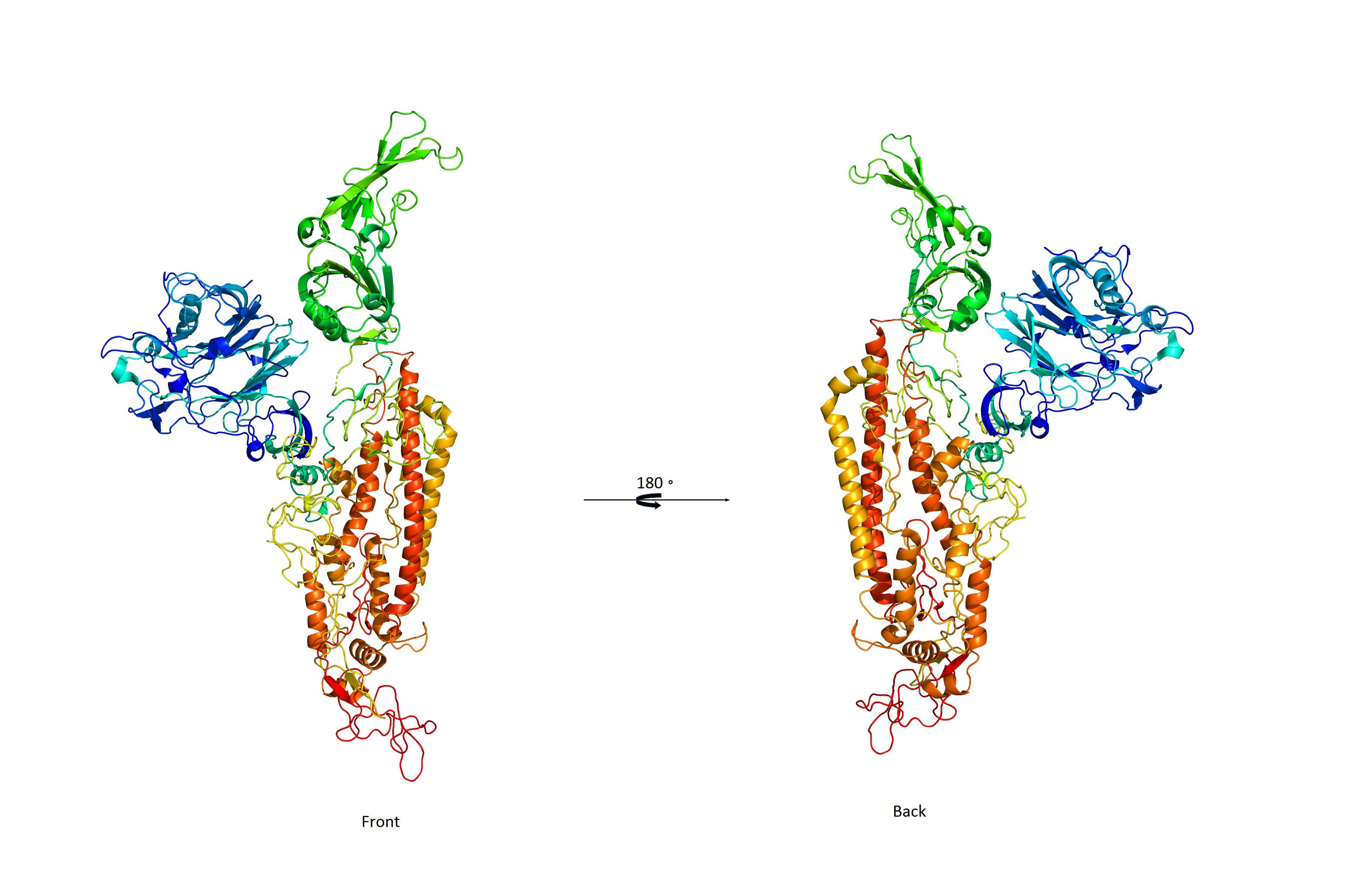


**Figure S2**. The 3D structure of the MERS-COV S protein (front and back conformations).


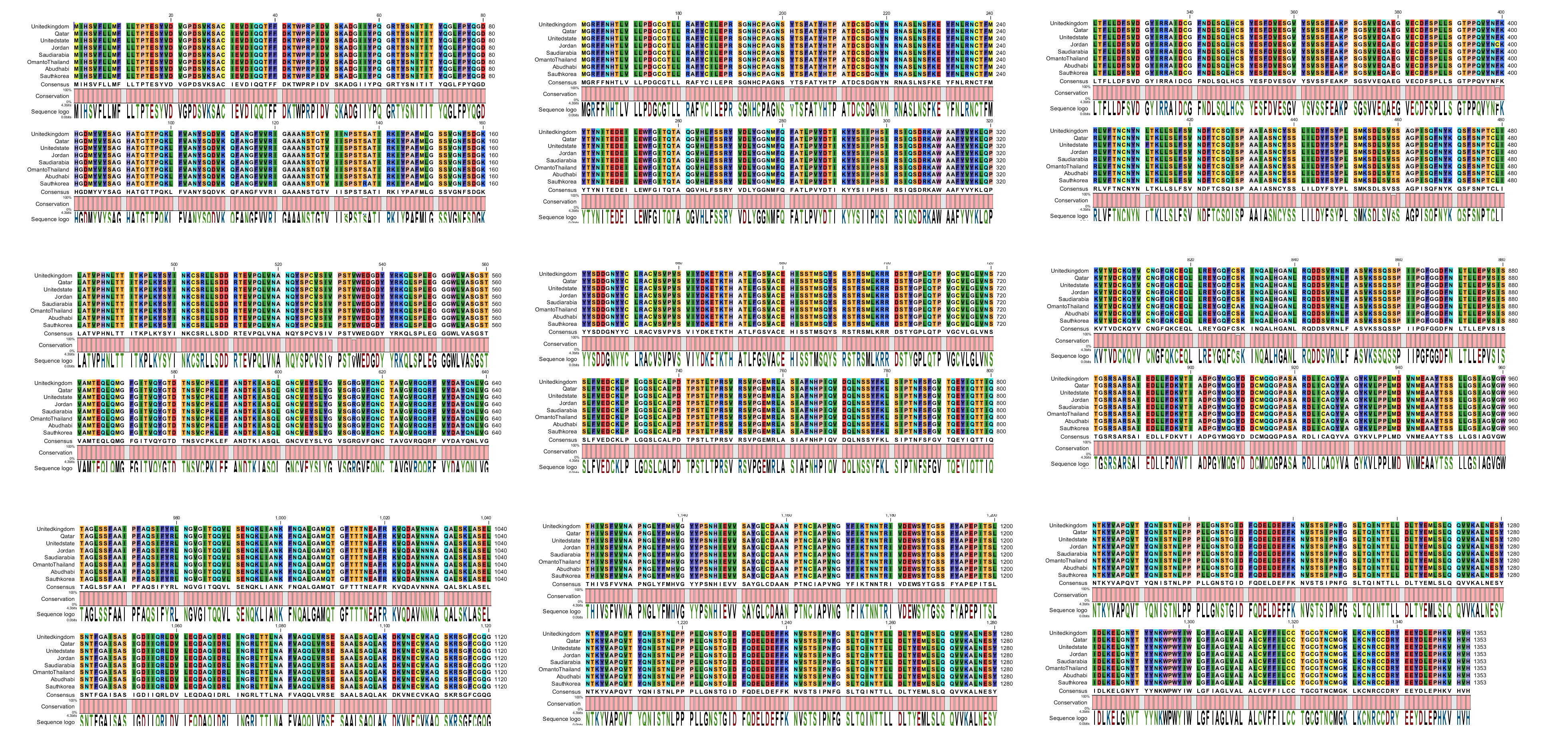


**Figure S3**. Multiple sequence alignment showing conservation of the S protein of MERS-COV isolated from eight different countries.


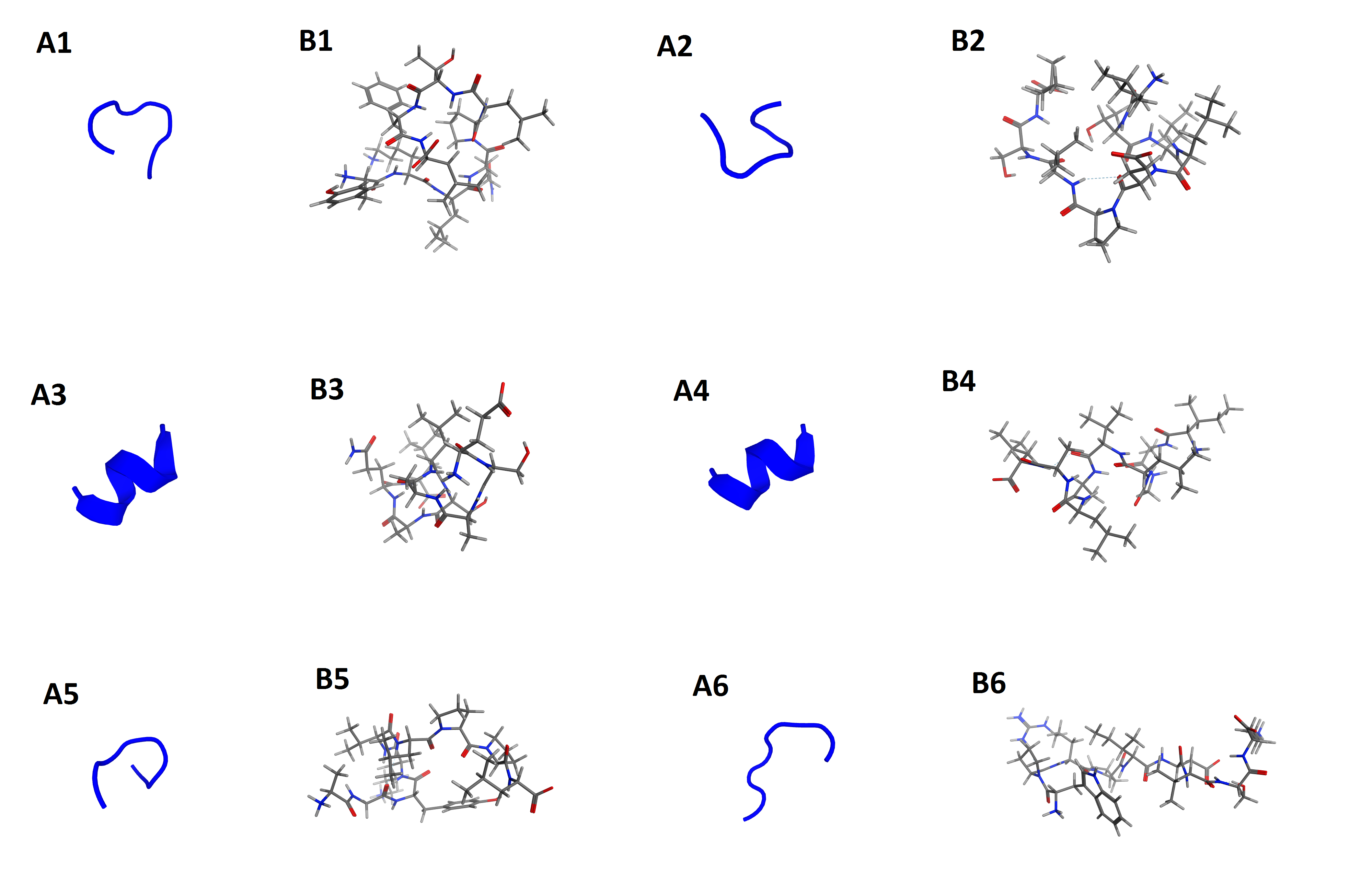


**Figure S4**. 3D (A1-6) and stick structures (B1-6) representation of selected MHC class-I alleles binding peptides. The figure is in symmetry with the information provided in Table 3.


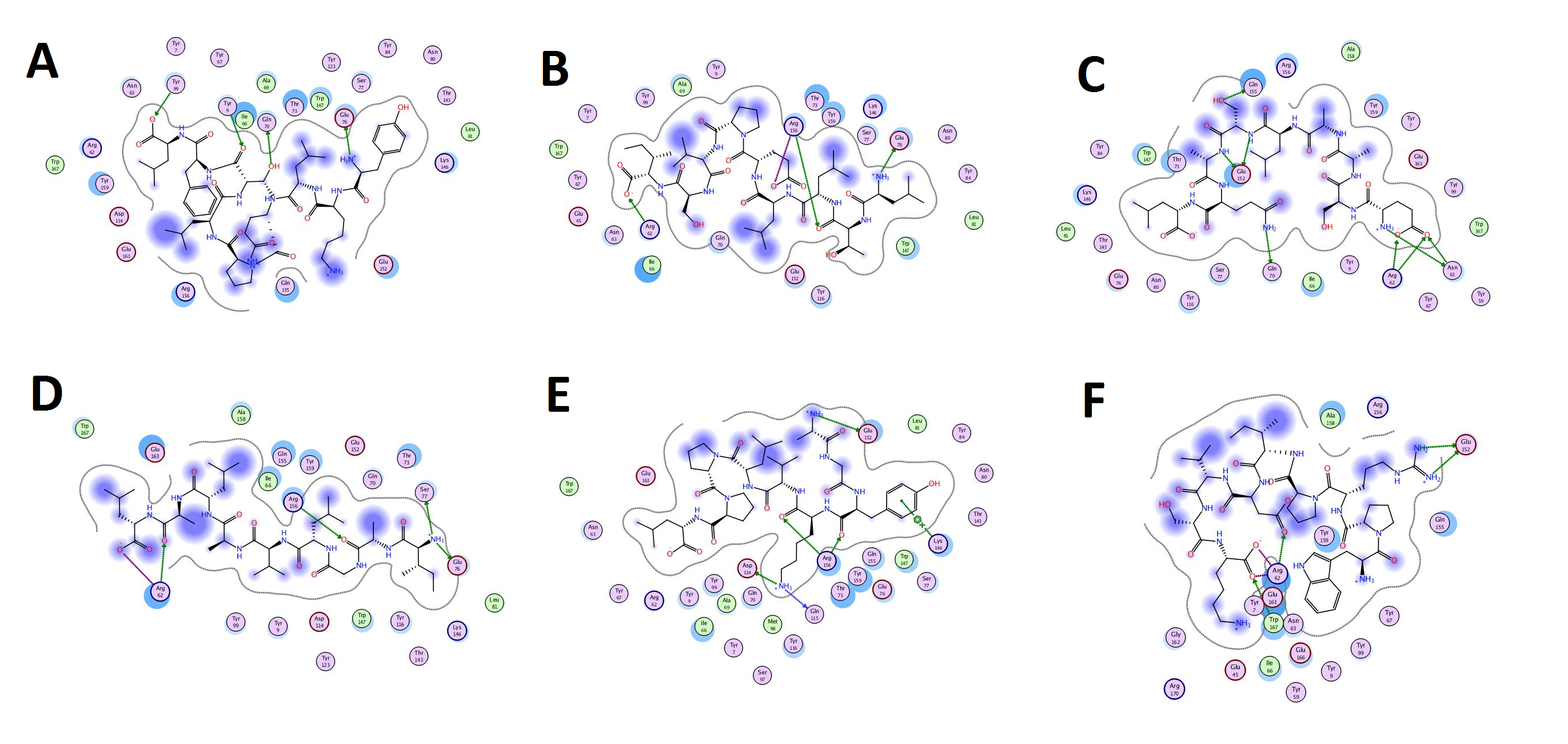


**Figure S5**. 2D graphical representation of interaction analyses between human HLA-B7 protein and MHC class-I alleles binding peptides. The figure is in symmetry with the information provided in Table 6 and Figure 6 and showing the residues interacting with strong hydrogen bonding.
