# Supplementary material for: Epitope‐based peptide vaccine design and target site depiction against Middle East Respiratory Syndrome Coronavirus: an immune-informatics study
Source: J Transl Med. 2019 Nov 8;17:362. doi: 10.1186/s12967-019-2116-8 (PMC6839065; doi:10.1186/s12967-019-2116-8)
Supplement: Supplementary file 2 — Additional file 2: Table S1. Physico-Chemical parameters of spike (S) protein computed through ExPASy ProtParam server. Table S2. Predicted disulphide bonds within residues of S protein via DiANNA 1.1 web Server. The bonds with lowest Score indicated as red colours are weak bonds. Table S3. Emini surface accessibility prediction results computed through IEDB Analysis Resource. Table S4. Conservancy results of B-cells and T-cells (MHC Class-I and II) epitopes among all 8 MERS-CoV isolates of distinct countries (Saudi Arabia, Abu Dhabi, Jordan, South Korea, Qatar, Thailand, USA and UK) have been shown. The analyses were done utilizing the IEDB Analysis Resource. [file 12967_2019_2116_MOESM2_ESM.docx]

**Table S1:** Physico-Chemical parameters of spike (S) protein computed through ExPASy ProtParam server**.**

| Parameters | S protein |
| --- | --- |
| Mol. Weight | 149368.04 Dalton |
| No. of amino acids | 1353 |
| Theoretical *pI* | 5.70 |
| Instability index (II) | 36.60 (stable) |
| No. of Negatively Charged Residues (Asp + Glu) | 112 |
| No. of Positively Charged Residues (Arg + Lys) | 95 |
| Aliphatic Index | 82.71 |
| Grand average of Hydropathicity (GRAVY) | -0.074 |
| Atomic Composition | \| Carbon \| 6682 \| \| --- \| --- \| \| Hydrogen \| 10245 \| \| Nitrogen \| 1735 \| \| Oxygen \| 2029 \| \| Sulfur \|  \| |
| Amino Acid Composition | \| Ala (A) \| 88 \| 6.5% \| \| --- \| --- \| --- \| \| Arg (R) \| 44 \| 3.3% \| \| Asn (N) \| 77 \| 5.7% \| \| Asp (D) \| 66 \| 4.9% \| \| Cys (C) \| 42 \| 3.1% \| \| Gln (Q) \| 72 \| 5.3% \| \| Glu (E) \| 46 \| 3.4% \| \| Gly (G) \| 92 \| 6.8% \| \| His (H) \| 20 \| 1.5% \| \| Ile (I) \| 73 \| 5.4% \| \| Leu (L) \| 120 \| 8.9% \| \| Lys (K) \| 51 \| 3.8% \| \| Met (M) \| 21 \| 1.6% \| \| Phe (F) \| 71 \| 5.2% \| \| Pro (P) \| 62 \| 4.6% \| \| Ser (S) \| 134 \| 9.9% \| \| Thr (T) \| 92 \| 6.8% \| \| Trp (W) \| 10 \| 0.7% \| \| Tyr (Y) \| 76 \| 5.6% \| \| Val (V) \| 96 \| 7.1% \| \| Pyl (O) \| 0 \| 0% \| \| Sec (U) \| 0 \| 0% \| |

**Table S2:** Predicted disulphide bonds within residues of spike protein via DiANNA 1.1 web

Server. The bonds with lowest Score indicated as red colours are weak bonds.

| Serial no positions peptide bonds scores |
| --- |
| 1 30-1106 SVKSACIEVDI-DKVNECVKAQS 0.96107 |
| 2 176-1319 LLPDGCGTLLR-VFFILCCTGCG 0.0104 |
| 3 185-817 LRAFYCILEPR-NGFQKCEQLLR 0.01042 |
| 4 195-237 RSGNHCPAGNS-FNLRNCTFMYT 0.72506 |
| 5 214-713 TPATDCSDGNY-QTPVGCVLGLV 0.99878 |
| 6 339-650 RRAIDCGFNDL-DGNYYCLRACV 0.98513 |
| 7 349-383 LSQLHCSYESF-AEGVECDFSPL 0.99964 |
| 8 407-1320 LVFTNCNYNLT-FFILCCTGCGT 0.01176 |
| 9 425-828 VNDFTCSQISP-EYGQFCSKINQ 0.95301 |
| 10 437-503 AIASNCYSSLI-SYINKCSRLLS 0.85147 |
| 11 478-620 FSNPTCLILAT-GVFQNCTAVGV 0.95997 |
| 12 526-654 NQYSPCVSILP-YCLRACVSVPV 0.9526 |
| 13 585-736 DTNSVCPKLEF-LGQSLCALPDT 0.98408 |
| 14 603-1117 SQLGNCVEYSL-KRSGFCGQGTH 0.99715 |
| 15 679-727 FGSVACEHISS-LFVEDCKLPLG 0.88581 |
| 16 806-811 KVTVDCKQYVC-CKQYVCNGFQK 0.99595 |
| 17 912-1164 QGYDDCMQQGP-ANPTNCIAPVN 0.43292 |
| 18 925-1337 ARDLICAQYVA-KCNRCCDRYEE 0.99536 |
| 19 1156-1336 SAYGLCDAANP-LKCNRCCDRYE 0.98674 |
| 20 1313-1327 VALALCVFFIL-GCGTNCMGKLK 0.01058 |
| 21 1323-1333 LCCTGCGTNCM-MGKLKCNRCCD 0.99851 |

**Table S3:** Emini surface accessibility prediction results computed through IEDB Analysis Resource**.**

| Position Residue Start End Peptide Score |
| --- |
| 306 D 304 309 QSDRKA 4.076 |
| 468 N 466 471 QFNYKQ 3.478 |
| 510 D 508 513 SDDRTE 4.862 |
| 540 Y 538 543 GDYYRK 4.224 |
| 541 Y 539 544 DYYRKQ 7.392 |
| 542 R 540 545 YYRKQL 3.65 |
| 665 K 663 668 YDKETK 6.951 |
| 666 E 664 669 DKETKT 6.403 |
| 667 T 665 670 KETKTH 5.217 |
| 690 S 688 693 QYSRST 3.661 |
| 691 R 689 694 YSRSTR 4.14 |
| 692 S 690 695 SRSTRS 3.541 |
| 699 R 697 702 LKRRDS 3.763 |
| 700 R 698 703 KRRDST 6.585 |
| 701 D 699 704 RRDSTY 5.16 |
| 1102 K 1100 1105 KDKVNE 3.669 |
| 1110 Q 1108 1113 KAQSKR 4.881 |
| 1176 N 1174 1179 KTNNTR 5.607 |
| 1290 T 1288 1293 NYTYYN 3.816 |
| 1291 Y 1289 1294 YTYYNK 4.745 |
| 1340 Y 1338 1343 DRYEEY 6.401 |
| 1341 E 1339 1344 RYEEYD 6.401 |

**Table S4:** Conservancy results of B-cells and T-cells (MHC Class-I and II) epitopes among all 8 MERS-CoV isolates of different countries (Saudi Arabia, Abu Dhabi, Jordan, Qatar, South Korea, Thailand, USA and UK) have been shown. The analyses were done using the IEDB Analysis Resource.

| Sr# | Sequences | Epitope length | Identity (8/8) |
| --- | --- | --- | --- |
| B-Cells peptides | | | |
| 1 | TPTESYVDVGPDSV | 14 | 100% |
| 2 | TPATDCSDGNYNRN | 14 | 100% |
| 3 | LEWFGITQTAQGVH | 14 | 100% |
| 4 | QLQMGFGITVQYGT | 14 | 100% |
| 5 | GNYTYYNKWPWYIW | 14 | 100% |
| 6 | RYEEYDLEPHKVHV | 14 | 100% |
| MHC class-I binding peptides | | | |
| 1 | YKLQPLTFL | 9 | 100% |
| 2 | LTLLEPVSI | 9 | 100% |
| 3 | ESAALSAQL | 9 | 100% |
| 4 | IAGLVALAL | 9 | 100% |
| 5 | AGYKVLPPL | 9 | 100% |
| 6 | WPRPIDVSK | 9 | 100% |
| MHC class-II binding peptides | | | |
| 1 | YCILEPRSG | 9 | 100% |
| 2 | LYFMHVGYY | 9 | 100% |
| 3 | MRLASIAFN | 9 | 100% |
| 4 | FGITQTAQG | 9 | 100% |
| 5 | VRIGAAANS | 9 | 100% |
| 6 | VYKLQPLTF | 9 | 100% |
